# 1-(4-{[(*E*)-3-Eth­oxy-2-hy­droxy­benzyl­idene]amino}­phen­yl)ethanone oxime

**DOI:** 10.1107/S1600536812001213

**Published:** 2012-01-18

**Authors:** Li Zhao, Xu-Tao Dong, Si-Jia Xing, Qian Cheng, Ji-Xing Zhao

**Affiliations:** a School of Chemical and Biological Engineering, Lanzhou Jiaotong University, Lanzhou 730070, People’s Republic of China

## Abstract

In the title compound, C_17_H_18_N_2_O_3_, the benzene rings form a dihedral angle of 3.34 (2)°. There is a strong intra­molecular O—H⋯N hydrogen bonds (which induces planarity of the structure). In the crystal, mol­ecules are linked by pairs of O—H⋯N hydrogen bonds, forming inversion dimers.

## Related literature

For background to oxime-type compounds, see: Dong *et al.*, (2009 ▶); Narasaka & Kitamura (2005 ▶). For their syntheses and structures, see: Dong *et al.* (2008 ▶); Akine *et al.* (2002 ▶); Wu *et al.* (2010 ▶).
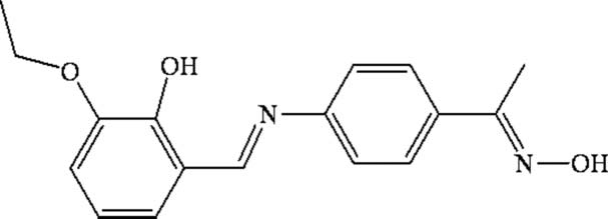


## Experimental

### 

#### Crystal data

C_17_H_18_N_2_O_3_
*M* *_r_* = 298.33
Triclinic, 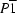

*a* = 7.0556 (7) Å
*b* = 7.4852 (9) Å
*c* = 14.7821 (16) Å
α = 96.890 (1)°
β = 98.762 (1)°
γ = 102.105 (2)°
*V* = 745.03 (14) Å^3^
*Z* = 2
Mo *K*α radiation
μ = 0.09 mm^−1^
*T* = 298 K
0.32 × 0.21 × 0.13 mm

#### Data collection

Bruker SMART 1000 CCD area-detector diffractometer
Absorption correction: multi-scan (*SADABS*; Sheldrick, 1996 ▶) *T*_min_ = 0.971, *T*_max_ = 0.988
3664 measured reflections
2561 independent reflections
1376 reflections with *I* > 2σ(*I*)
*R*_int_ = 0.037

#### Refinement

*R*[*F* ^2^ > 2σ(*F* ^2^)] = 0.068
*wR*(*F* ^2^) = 0.168
*S* = 1.02
2561 reflections
201 parameters
H-atom parameters constrained
Δρ_max_ = 0.19 e Å^−3^
Δρ_min_ = −0.27 e Å^−3^

### 

Data collection: *SMART* (Siemens, 1996 ▶); cell refinement: *SAINT* (Siemens, 1996 ▶); data reduction: *SAINT*; program(s) used to solve structure: *SHELXS97* (Sheldrick, 2008 ▶); program(s) used to refine structure: *SHELXL97* (Sheldrick, 2008 ▶); molecular graphics: *SHELXTL* (Sheldrick, 2008 ▶); software used to prepare material for publication: *SHELXTL*.

## Supplementary Material

Crystal structure: contains datablock(s) global, I. DOI: 10.1107/S1600536812001213/hg5155sup1.cif

Structure factors: contains datablock(s) I. DOI: 10.1107/S1600536812001213/hg5155Isup2.hkl

Supplementary material file. DOI: 10.1107/S1600536812001213/hg5155Isup3.cml

Additional supplementary materials:  crystallographic information; 3D view; checkCIF report

## Figures and Tables

**Table 1:** Hydrogen-bond geometry (Å, °)

*D*—H⋯*A*	*D*—H	H⋯*A*	*D*⋯*A*	*D*—H⋯*A*
O1—H1⋯N1^i^	0.82	2.07	2.817 (4)	152
O2—H2⋯N2	0.82	1.84	2.567 (3)	147

Symmetry code: (i) 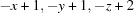.

**Fig. 1.**
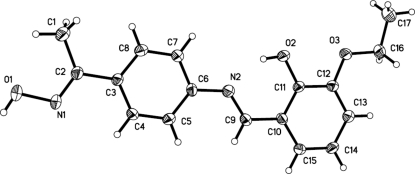
The molecule structure of the title compound with atom numbering scheme. Displacement ellipsoids for non-hydrogen atoms are drawn at the 30% probability level.

**Fig. 2.**
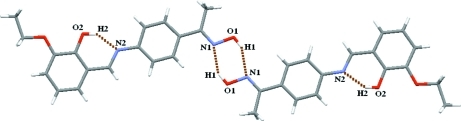
Digram showing the intramolecular O—H···N and intermolecular O—H···N hydrogen bonding interactions. Hydrogen atoms not involved have been deleted for clarity.

## supplementary crystallographic information

### Comment

Oxime-type compounds are a traditional class of chelating ligands widely used in coordination and analytical chemistry and extraction metallurgy (Dong *et al.*, 2009; Narasaka *et al.*, 2005). In the last few years, a large number of oxime-type compounds and their complexes have been reported (Dong *et al.*, 2008; Akine *et al.*, 2002). However, the oxime-type compounds derived the 4-aminophenylethanone have never been reported. In this paper, the crystal structure of new oxime-type compound, 1-(4-{[(*E*)-3-ethoxy-2-hydroxybenzylidene] amino}phenyl)ethanone oxime, derived from the reaction of 4-amino-phenylethanone oxime and 3-ethoxysalicylaldehyde (I), (Fig. 1) is reported.

The single-crystal structure of the title compound was determined by X-ray crystallography. The crystal structure of the title compound is only built up by the C_17_H_18_N_2_O_3_ molecules, in which all bond lengths are in normal ranges. The two benzene rings form a dihedral angle of 3.34 (2) °. There is a strong intramolecular O2–H2···N2 hydrogen bonds (which induces planarity on the structure). In the crystal structure, the molecules form dimers disposed about two pairs of inter-molecular O—H..N hydrogen bonds. (Table 1)(Wu *et al.*, 2010).

### Experimental

To an ethanol solution (5 ml) of 3-ethoxysalicylaldehyde(166.2 mg, 1.00 mmol) was added an ethanol solution (5 ml) of 4-aminophenylethanone oxime (151.7 mg, 1.00 mmol). After the solution had been stirred at 328 K for 5 h, the mixture was filtered. The residue was washed successively with ethanol and n-hexane, respectively. The isolated compound was dried under reduced pressure to yield 281.1 mg of yellow solid (yield 80%, m.p. 436–437 K). Elemental analysis also supports composition of the title compound. Anal. calcd. for C_17_H_18_N_2_O_3_: C 68.44; H 6.08; N 9.39; Found: C, 68.30; H, 6.02; N, 9.52. The single crystals were obtained by slow evaporation from an acetonitrile solution at room temperature.

### Refinement

H atoms were treated as riding atoms with distances C—H = 0.96 (CH_3_), C—H = 0.97 (CH_2_), 0.93 Å (CH), 0.82 Å (OH), and the values of *U*_iso_(H) for the thermal parameters for aromatic, methylene and hydroxy protons *U*_iso_(H) = 1.2 *U*_eq_(C)(aromatic), *U*_iso_(H) = 1.5 *U*_eq_(*C*)(methylene) and *U*_iso_(H) = 1.5 *U*_eq_(*C*)(hydroxy), respectively.

### Crystal data

**Table:**

C_17_H_18_N_2_O_3_	*Z* = 2
*M**_r_* = 298.33	*F*(000) = 316
Triclinic, *P*1	*D*_x_ = 1.330 Mg m^−^^3^
Hall symbol: -P 1	Mo *K*α radiation, λ = 0.71073 Å
*a* = 7.0556 (7) Å	Cell parameters from 818 reflections
*b* = 7.4852 (9) Å	θ = 2.8–27.5°
*c* = 14.7821 (16) Å	µ = 0.09 mm^−^^1^
α = 96.890 (1)°	*T* = 298 K
β = 98.762 (1)°	Prismatical, yellow
γ = 102.105 (2)°	0.32 × 0.21 × 0.13 mm
*V* = 745.03 (14) Å^3^	

### Data collection

**Table:**

Bruker SMART 1000 CCD area-detector diffractometer	2561 independent reflections
Radiation source: fine-focus sealed tube	1376 reflections with *I* > 2σ(*I*)
graphite	*R*_int_ = 0.037
phi and ω scans	θ_max_ = 25.0°, θ_min_ = 2.8°
Absorption correction: multi-scan (*SADABS*; Sheldrick, 1996)	*h* = −8→8
*T*_min_ = 0.971, *T*_max_ = 0.988	*k* = −8→8
3664 measured reflections	*l* = −17→9

### Refinement

**Table:**

Refinement on *F*^2^	Primary atom site location: structure-invariant direct methods
Least-squares matrix: full	Secondary atom site location: difference Fourier map
*R*[*F*^2^ > 2σ(*F*^2^)] = 0.068	Hydrogen site location: inferred from neighbouring sites
*wR*(*F*^2^) = 0.168	H-atom parameters constrained
*S* = 1.02	*w* = 1/[σ^2^(*F*_o_^2^) + (0.0613*P*)^2^ + 0.2977*P*] where *P* = (*F*_o_^2^ + 2*F*_c_^2^)/3
2561 reflections	(Δ/σ)_max_ < 0.001
201 parameters	Δρ_max_ = 0.19 e Å^−^^3^
0 restraints	Δρ_min_ = −0.27 e Å^−^^3^

### Special details

**Table:**

Geometry. All e.s.d.'s (except the e.s.d. in the dihedral angle between two l.s. planes) are estimated using the full covariance matrix. The cell e.s.d.'s are taken into account individually in the estimation of e.s.d.'s in distances, angles and torsion angles; correlations between e.s.d.'s in cell parameters are only used when they are defined by crystal symmetry. An approximate (isotropic) treatment of cell e.s.d.'s is used for estimating e.s.d.'s involving l.s. planes.
Refinement. Refinement of *F*^2^ against ALL reflections. The weighted *R*-factor *wR* and goodness of fit *S* are based on *F*^2^, conventional *R*-factors *R* are based on *F*, with *F* set to zero for negative *F*^2^. The threshold expression of *F*^2^ > σ(*F*^2^) is used only for calculating *R*-factors(gt) *etc*. and is not relevant to the choice of reflections for refinement. *R*-factors based on *F*^2^ are statistically about twice as large as those based on *F*, and *R*- factors based on ALL data will be even larger.

### Fractional atomic coordinates and isotropic or equivalent isotropic displacement parameters (Å^2^)

**Table:**

	*x*	*y*	*z*	*U*_iso_*/*U*_eq_	
N1	0.3517 (4)	0.5142 (4)	0.91196 (19)	0.0451 (8)	
N2	0.4699 (4)	0.7402 (4)	0.51348 (18)	0.0399 (7)	
O1	0.2676 (4)	0.4614 (4)	0.98854 (17)	0.0644 (8)	
H1	0.3548	0.4525	1.0301	0.097*	
O2	0.3342 (3)	0.8040 (3)	0.35234 (16)	0.0518 (7)	
H2	0.3308	0.7783	0.4046	0.078*	
O3	0.3898 (3)	0.8888 (4)	0.19161 (16)	0.0522 (7)	
C1	0.0075 (5)	0.5044 (6)	0.8478 (3)	0.0667 (13)	
H1A	−0.0132	0.4861	0.9090	0.100*	
H1B	−0.0658	0.3980	0.8041	0.100*	
H1C	−0.0362	0.6120	0.8325	0.100*	
C2	0.2226 (5)	0.5310 (5)	0.8443 (2)	0.0396 (9)	
C3	0.2949 (5)	0.5820 (4)	0.7593 (2)	0.0332 (8)	
C4	0.4926 (5)	0.6169 (5)	0.7524 (2)	0.0443 (10)	
H4	0.5844	0.6042	0.8020	0.053*	
C5	0.5573 (5)	0.6699 (5)	0.6740 (2)	0.0465 (10)	
H5	0.6913	0.6937	0.6720	0.056*	
C6	0.4246 (5)	0.6879 (4)	0.5984 (2)	0.0351 (8)	
C7	0.2268 (5)	0.6483 (5)	0.6030 (2)	0.0446 (10)	
H7	0.1352	0.6561	0.5522	0.053*	
C8	0.1624 (5)	0.5971 (5)	0.6820 (2)	0.0449 (10)	
H8	0.0282	0.5724	0.6836	0.054*	
C9	0.6452 (5)	0.7803 (4)	0.4955 (2)	0.0376 (9)	
H9	0.7514	0.7766	0.5404	0.045*	
C10	0.6814 (5)	0.8313 (4)	0.4067 (2)	0.0344 (8)	
C11	0.5221 (5)	0.8398 (4)	0.3389 (2)	0.0355 (8)	
C12	0.5558 (5)	0.8854 (5)	0.2518 (2)	0.0382 (9)	
C13	0.7458 (5)	0.9199 (5)	0.2339 (2)	0.0412 (9)	
H13	0.7682	0.9488	0.1764	0.049*	
C14	0.9048 (5)	0.9119 (5)	0.3015 (2)	0.0427 (9)	
H14	1.0321	0.9360	0.2890	0.051*	
C15	0.8720 (5)	0.8682 (5)	0.3863 (2)	0.0384 (9)	
H15	0.9781	0.8630	0.4309	0.046*	
C16	0.4125 (5)	0.9423 (5)	0.1031 (2)	0.0506 (10)	
H16A	0.4586	0.8501	0.0659	0.061*	
H16B	0.5070	1.0601	0.1109	0.061*	
C17	0.2127 (6)	0.9575 (7)	0.0572 (3)	0.0781 (15)	
H17A	0.1228	0.8383	0.0466	0.117*	
H17B	0.2220	1.0006	−0.0009	0.117*	
H17C	0.1655	1.0433	0.0966	0.117*	

### Atomic displacement parameters (Å^2^)

**Table:**

	*U*^11^	*U*^22^	*U*^33^	*U*^12^	*U*^13^	*U*^23^
N1	0.0459 (19)	0.059 (2)	0.0336 (18)	0.0094 (15)	0.0162 (14)	0.0153 (14)
N2	0.0371 (18)	0.0485 (19)	0.0364 (18)	0.0112 (14)	0.0100 (13)	0.0084 (14)
O1	0.0549 (18)	0.103 (2)	0.0438 (17)	0.0163 (16)	0.0224 (13)	0.0324 (15)
O2	0.0305 (15)	0.085 (2)	0.0447 (16)	0.0123 (13)	0.0110 (11)	0.0243 (13)
O3	0.0358 (15)	0.085 (2)	0.0345 (15)	0.0073 (13)	0.0001 (11)	0.0229 (13)
C1	0.043 (3)	0.111 (4)	0.048 (3)	0.014 (2)	0.0157 (19)	0.018 (2)
C2	0.038 (2)	0.046 (2)	0.033 (2)	0.0060 (17)	0.0095 (16)	0.0061 (17)
C3	0.033 (2)	0.037 (2)	0.031 (2)	0.0085 (15)	0.0097 (14)	0.0051 (15)
C4	0.034 (2)	0.071 (3)	0.032 (2)	0.0155 (18)	0.0049 (15)	0.0166 (19)
C5	0.031 (2)	0.071 (3)	0.043 (2)	0.0136 (19)	0.0118 (17)	0.0162 (19)
C6	0.042 (2)	0.038 (2)	0.030 (2)	0.0128 (17)	0.0108 (15)	0.0097 (16)
C7	0.037 (2)	0.063 (3)	0.038 (2)	0.0144 (19)	0.0075 (16)	0.0171 (19)
C8	0.032 (2)	0.062 (3)	0.044 (2)	0.0126 (18)	0.0096 (16)	0.0113 (19)
C9	0.038 (2)	0.043 (2)	0.032 (2)	0.0111 (17)	0.0029 (15)	0.0089 (16)
C10	0.035 (2)	0.039 (2)	0.030 (2)	0.0110 (16)	0.0047 (14)	0.0077 (15)
C11	0.030 (2)	0.044 (2)	0.035 (2)	0.0091 (16)	0.0106 (15)	0.0081 (16)
C12	0.032 (2)	0.045 (2)	0.034 (2)	0.0044 (17)	0.0019 (15)	0.0063 (16)
C13	0.038 (2)	0.046 (2)	0.040 (2)	0.0087 (17)	0.0090 (16)	0.0100 (17)
C14	0.031 (2)	0.051 (2)	0.047 (2)	0.0067 (17)	0.0134 (16)	0.0093 (18)
C15	0.031 (2)	0.049 (2)	0.036 (2)	0.0109 (17)	0.0047 (15)	0.0102 (17)
C16	0.054 (3)	0.060 (3)	0.037 (2)	0.009 (2)	0.0047 (17)	0.0163 (19)
C17	0.064 (3)	0.110 (4)	0.059 (3)	0.013 (3)	−0.003 (2)	0.038 (3)

### Geometric parameters (Å, °)

**Table:**

N1—C2	1.283 (4)	C7—C8	1.383 (5)
N1—O1	1.416 (3)	C7—H7	0.9300
N2—C9	1.286 (4)	C8—H8	0.9300
N2—C6	1.420 (4)	C9—C10	1.454 (4)
O1—H1	0.8200	C9—H9	0.9300
O2—C11	1.346 (4)	C10—C15	1.401 (4)
O2—H2	0.8200	C10—C11	1.406 (4)
O3—C12	1.366 (4)	C11—C12	1.411 (4)
O3—C16	1.435 (4)	C12—C13	1.384 (4)
C1—C2	1.498 (5)	C13—C14	1.400 (4)
C1—H1A	0.9600	C13—H13	0.9300
C1—H1B	0.9600	C14—C15	1.374 (4)
C1—H1C	0.9600	C14—H14	0.9300
C2—C3	1.488 (4)	C15—H15	0.9300
C3—C4	1.387 (4)	C16—C17	1.502 (5)
C3—C8	1.393 (4)	C16—H16A	0.9700
C4—C5	1.379 (5)	C16—H16B	0.9700
C4—H4	0.9300	C17—H17A	0.9600
C5—C6	1.384 (4)	C17—H17B	0.9600
C5—H5	0.9300	C17—H17C	0.9600
C6—C7	1.378 (5)		
			
C2—N1—O1	112.6 (3)	N2—C9—H9	119.2
C9—N2—C6	124.4 (3)	C10—C9—H9	119.2
N1—O1—H1	109.5	C15—C10—C11	119.0 (3)
C11—O2—H2	109.5	C15—C10—C9	121.4 (3)
C12—O3—C16	118.0 (3)	C11—C10—C9	119.6 (3)
C2—C1—H1A	109.5	O2—C11—C10	122.8 (3)
C2—C1—H1B	109.5	O2—C11—C12	117.3 (3)
H1A—C1—H1B	109.5	C10—C11—C12	119.9 (3)
C2—C1—H1C	109.5	O3—C12—C13	125.9 (3)
H1A—C1—H1C	109.5	O3—C12—C11	114.6 (3)
H1B—C1—H1C	109.5	C13—C12—C11	119.5 (3)
N1—C2—C3	117.0 (3)	C12—C13—C14	120.7 (3)
N1—C2—C1	123.4 (3)	C12—C13—H13	119.7
C3—C2—C1	119.5 (3)	C14—C13—H13	119.7
C4—C3—C8	116.9 (3)	C15—C14—C13	119.8 (3)
C4—C3—C2	122.9 (3)	C15—C14—H14	120.1
C8—C3—C2	120.2 (3)	C13—C14—H14	120.1
C5—C4—C3	122.0 (3)	C14—C15—C10	121.1 (3)
C5—C4—H4	119.0	C14—C15—H15	119.4
C3—C4—H4	119.0	C10—C15—H15	119.4
C4—C5—C6	120.6 (3)	O3—C16—C17	106.4 (3)
C4—C5—H5	119.7	O3—C16—H16A	110.5
C6—C5—H5	119.7	C17—C16—H16A	110.5
C7—C6—C5	118.2 (3)	O3—C16—H16B	110.5
C7—C6—N2	115.2 (3)	C17—C16—H16B	110.5
C5—C6—N2	126.6 (3)	H16A—C16—H16B	108.7
C6—C7—C8	121.1 (3)	C16—C17—H17A	109.5
C6—C7—H7	119.4	C16—C17—H17B	109.5
C8—C7—H7	119.4	H17A—C17—H17B	109.5
C7—C8—C3	121.2 (3)	C16—C17—H17C	109.5
C7—C8—H8	119.4	H17A—C17—H17C	109.5
C3—C8—H8	119.4	H17B—C17—H17C	109.5
N2—C9—C10	121.7 (3)		
			
O1—N1—C2—C3	−178.6 (3)	N2—C9—C10—C15	−177.6 (3)
O1—N1—C2—C1	2.1 (5)	N2—C9—C10—C11	0.9 (5)
N1—C2—C3—C4	−2.2 (5)	C15—C10—C11—O2	179.1 (3)
C1—C2—C3—C4	177.1 (3)	C9—C10—C11—O2	0.6 (5)
N1—C2—C3—C8	178.0 (3)	C15—C10—C11—C12	−0.1 (5)
C1—C2—C3—C8	−2.7 (5)	C9—C10—C11—C12	−178.6 (3)
C8—C3—C4—C5	2.0 (5)	C16—O3—C12—C13	−3.5 (5)
C2—C3—C4—C5	−177.8 (3)	C16—O3—C12—C11	177.2 (3)
C3—C4—C5—C6	−0.8 (6)	O2—C11—C12—O3	0.6 (4)
C4—C5—C6—C7	−1.2 (5)	C10—C11—C12—O3	179.8 (3)
C4—C5—C6—N2	−179.7 (3)	O2—C11—C12—C13	−178.8 (3)
C9—N2—C6—C7	−179.2 (3)	C10—C11—C12—C13	0.5 (5)
C9—N2—C6—C5	−0.6 (5)	O3—C12—C13—C14	−179.9 (3)
C5—C6—C7—C8	1.9 (5)	C11—C12—C13—C14	−0.6 (5)
N2—C6—C7—C8	−179.4 (3)	C12—C13—C14—C15	0.4 (5)
C6—C7—C8—C3	−0.7 (5)	C13—C14—C15—C10	0.0 (5)
C4—C3—C8—C7	−1.3 (5)	C11—C10—C15—C14	−0.1 (5)
C2—C3—C8—C7	178.6 (3)	C9—C10—C15—C14	178.4 (3)
C6—N2—C9—C10	179.5 (3)	C12—O3—C16—C17	−172.9 (3)

### Hydrogen-bond geometry (Å, °)

**Table:**

*D*—H···*A*	*D*—H	H···*A*	*D*···*A*	*D*—H···*A*
O1—H1···N1^i^	0.82	2.07	2.817 (4)	152
O2—H2···N2	0.82	1.84	2.567 (3)	147

Symmetry codes: (i) −*x*+1, −*y*+1, −*z*+2.
